# Production of selectable marker gene-free Cavendish banana (*Musa* spp.) using a steroid-inducible recombinase platform

**DOI:** 10.1007/s11248-019-00179-6

**Published:** 2019-10-29

**Authors:** Jennifer Kleidon, Anthony Brinin, Jean-Yves Paul, Robert Harding, James Dale, Benjamin Dugdale

**Affiliations:** Centre for Tropical Crops and Biocommodities, Queensland University of Technology, Brisbane, QLD 4000 Australia

**Keywords:** Cavendish, Banana, Marker-free, Recombinase

## Abstract

Genetic improvement of commercially accepted banana cultivars is strongly reliant on the ability to introduce genes that encode important agro-traits such as disease resistance. In most cases this can only be achieved using a transgenic approach. Public and regulatory acceptance of these events would greatly increase with “clean” single copy integration events free of the selectable marker gene and extraneous vector backbone. This would also allow for the successive addition of new genes and traits as they become available. In this study, we used the pMarker Free 1 (pMF1) vector containing the green fluorescent protein (*gfp*) reporter gene to assess the effectiveness of steroid-inducible recombination and positive/negative dual selection to regenerate transgenic Cavendish banana plants that were potentially free of the selectable marker gene. By examining the interaction of two different *Agrobacterium* strains with two different cultivars of Cavendish banana, namely Williams and Grand Naine, we describe a transformation and regeneration strategy that successfully produced marker-free, single transgene copy, *gfp*-expressing events. The system will provide a useful means of serially improving banana into the future.

## Introduction

A major challenge facing global banana production is the development of disease resistant varieties. Bananas (*Musa* spp.) are threatened by a range of diseases, the most devastating being Black Sigatoka caused by *Pseudocercospora fijiensis* (formerly *Mycosphaerella fijiensis*) (Arango Isaza et al. 2016), Panama disease or Fusarium wilt caused by *Fusarium oxysporum* f. sp. *cubense* (Ploetz 2015), banana *Xanthomonas* wilt caused by the bacterium *Xanthomonas campestris* pv. *musacearum* (Blomme et al. 2013) and banana bunchy top disease caused by *Banana bunchy top virus* (BBTV) (Dale 1987). Domesticated bananas, particularly the preferred AAA types (e.g. Cavendish), are essentially sterile making conventional breeding from current cultivars difficult. While breeding with seeded and fertile wild diploids has been successful, this process is extremely time consuming and often the new cultivars do not have the agronomic and fruit qualities required for commercial and public acceptance. Further, not all the desired resistance traits are available in the accessible banana gene pool. Molecular breeding of elite banana cultivars is, therefore, the most suitable strategy for the genetic improvement of current cultivars. Technologies that facilitate genetic modification and transgene expression in banana have advanced rapidly over the past twenty years. In this time, the development of efficient methods for plant transformation and regeneration (Khanna et al. 2004), advances in pathogen resistance strategies (Shekhawat et al. 2012; Tripathi et al. 2014; Dale et al. 2017) and sequencing of a diploid banana genome (D’Hont et al. 2012) have collectively made engineered disease resistance in banana an achievable goal.

The production of transgenic banana relies on the use of selectable marker genes, usually conferring antibiotic tolerance, to preferentially select transformed cells containing the gene of interest. Despite extensive evidence to support their biosafety, there remains a negative public perception regarding their integration into the host genome (Miki and McHugh 2004; Tuteja et al. 2012; Rosellini 2012). Furthermore, selectable marker genes serve no useful purpose after the initial selection phase. In conjunction with the pressures of population growth and climate change, exclusion of selectable marker genes is likely to increase the acceptance of genetically modified (GM) crops. In addition to public and regulatory concerns, the constitutive expression of selectable marker genes in GM plants may be a metabolic burden to the host and may negatively impact on important agronomic traits or the plant’s natural tolerance to environmental stresses (Yau and Stewart 2013). Importantly, their use also prevents the re-use of the same selectable marker gene when a second transformation scheme, serial transformation, is required with an elite transgenic host. This is of particular relevance to gene stacking strategies and the continuous improvement of banana and other crops against emerging disease threats or with other enhanced agronomic traits.

A number of approaches have been described to avoid or eliminate antibiotic/herbicide selectable marker genes from transgenic crops (reviewed in Miki and McHugh 2004; Tuteja et al. 2012). The marker-free system developed by Schaart et al. (2004) was based on the R-Rs recombinase from the yeast, *Zygosaccharomyces rouxii* and has been utilized effectively in the production of ‘cisgenic’ apple and strawberry, i.e. marker-free GM plants in which the gene of interest and the regulatory sequences controlling its expression are derived from a plant species that is sexually compatible with the host (Schaart et al. 2004; Vanblaere et al. 2011; Krens et al. 2015). In this system, the R recombinase is fused to a ligand binding domain (LBD), a steroid binding domain derived from a steroid hormone receptor protein. The R recombinase-LBD (R-LBD) gene fusion is constitutively expressed from a cassette located between repeats of the R recombinase recognition sequences. In the absence of the steroid inducer molecule, dexamethasone (DEX), the R-LBD fusion protein is sequestered in the cytoplasm by association with an abundant protein complex that includes heat shock protein 90. Addition of DEX results in the activation of the LBD and translocation of the R-LBD fusion protein to the nucleus with subsequent excision of DNA between the recombinase recognition sequences. Thus, recombination is induced only in the presence of the steroidal compound DEX. Also located between the recombination sites is an expression cassette containing the antibiotic selectable marker gene *npt*II fused to a positive selection gene, cytosine deaminase (*cod*A) from *Escherichia coli.* The *cod*A gene is a conditionally lethal dominant gene encoding an enzyme that converts the non-toxic 5-fluorocytosine (5-FC) to cytotoxic 5-fluorouracil (5-FU) (Mullen et al. 1992; Stougaard 1993). 5-FU is lethal to plant cells and, hence, those cells expressing *cod*A will die in the presence of 5-FC whereas those cells not expressing the enzyme (*cod*A has been excised) will survive. An expression cassette encoding the gene of interest is located on the T-DNA outside the recombination sites such that transgenic plants that have been successfully excised and selected on media containing both DEX and 5-FC contain only the transgene, recombination footprint and T-DNA integration borders.

In this study, we used the R-LBD recombinase system to generate selectable marker gene-free, GFP-transgenic Cavendish banana. Using two strains of *Agrobacterium tumefaciens* and two cultivars of Cavendish banana we describe a protocol that uses kanamycin to select for transgenic events and incorporates DEX-mediated recombination and 5-FC based negative selection for the complete removal of the antibiotic selection gene. GFP-expressing banana plants generated with this system were genotypically characterized for precise excision and recombination, extraneous vector backbone integration and transgene copy number. The outcome was an efficient excision platform for the production of low copy number, marker-free transgenic banana plants.

## Methods and materials

### Assembly of the marker-free vector

**Fig. 1:**
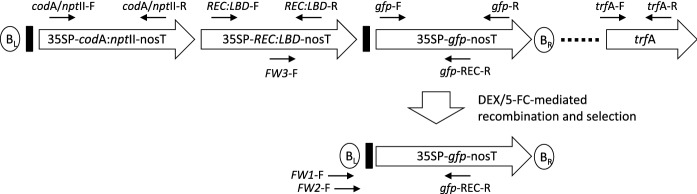
Schematic representation of the pMF1-GFP cassette pre- and post-recombinase-mediated excision and location of primers used for genotyping. 35SP = CaMV 35S promoter; *cod*A*:npt*II = *cytosine deaminase* A: *neomycin phosphotransferase* II gene fusion; nosT = nopaline synthase terminator; *REC:LBD* = *recombinase:ligand binding domain* gene fusion; *gfp* = *green fluorescent protein* reporter gene; *trf*A **= ***plasmid replication initiator protein* gene; B_L_ = left T-DNA border; B_R_ = right T-DNA border. Black rectangles represent the recombination footprint. Dotted lines represent vector backbone sequence. Primers used for genotyping are marked as thin arrows. Diagram not to scale

Vector pMF1 was kindly supplied by J. Schaart (Plant Research International, Wageningen). An expression cassette consisting of the CaMV 35S promoter (CaMV35SP), green fluorescent protein (*gfp*) reporter gene and nopaline synthase terminator (nosT) was PCR amplified from vector p35S-GFP with primers 35SP-F-Asc (5′-GGCGCGCCTCATGGAGTCAAAGATTCAAATAGAGG-3′) and nosT-R-Pac (5′-ATTAATTAAGAATTCGATTCTCGAGCCCGATCTAGTAACATAGATGACACCGCGC-3′) using the GoTaq^®^ Green PCR Master Mix system (Promega) and standard cycling conditions. The amplicon was cloned into pGEM^®^-T Easy vector (Promega) and verified using Sanger sequencing by Macrogen Inc. (South Korea). The expression cassette was subsequently ligated into pMF1 using unique AscI and PacI restriction sites to generate pMF1-GFP (Fig. 1). Vector pMF1-GFP was mobilized into *A. tumefaciens* strains LBA4404 and AGL1 by electroporation for banana transformation.

### *Agrobacterium*-mediated transformation of banana cells

*M. acuminata* cv. Cavendish (AAA subgroup) cultivars “Williams” and “Grand Naine” embryogenic cell suspensions were prepared from immature male flowers and *Agrobacterium*-mediated transformation was performed using the protocol described by Khanna et al. (2004), except cells were not centrifuged during co-cultivation with the bacteria. Following transformation, cells were layered on glass fibre filter disks and maintained on BL solid media (25–100 mg/L kanamycin; 200 mg/L cefotaxime) for 3 months with monthly sub-culturing. During this 3 month period, kanamycin concentrations were sequentially increased from 25 mg/L (month 1), to 50 mg/L (month 2) and finally 100 mg/L (month 3). Pre-embryogenic cells on filter disks were then transferred to solid M3 media (100 mg/L kanamycin; 200 mg/L cefotaxime) for 3 months with monthly sub-culturing. Plants were regenerated from embryos in M4 media and rooted in MS media (both containing 100 mg/L kanamycin and 200 mg/L cefotaxime). All tissue culture media constituents are described in Khanna et al. (2004).

### DEX and 5-FC treatments for removal of the selectable marker gene

Similar to standard transformation, banana cells transformed with pMF1-GFP were layered on glass fibre filter disks, and maintained on BL solid media (25–100 mg/L kanamycin; 200 mg/L cefotaxime) for 3 months with monthly sub-culturing. Cells were then harvested and the cell population from a single plate transferred to individual 50 mL Falcon tubes containing liquid BL media (100 µM DEX; 200 mg/L cefotaxime) without antibiotic selection. Cells were incubated for 5 days (LBA4404 transformations) or 10 days (AGL1 transformations) with gentle shaking. After DEX treatment, the cells were allowed to settle and resuspended in 1 mL of liquid BL media, pipetted onto a single glass fibre filter disk and maintained on solid M3 media (150 mg/L 5-FC; 1 µM DEX; 200 mg/L cefotaxime) without antibiotic selection, for 3 months with monthly sub-culturing. Plants were regenerated from embryos in M4 media (150 mg/L 5-FC; 1 µM DEX; 200 mg/L cefotaxime) and rooted in MS media (150 mg/L 5-FC; 200 mg/L cefotaxime). A single plant representing each plate was selected for genotyping.

### PCR genotyping

Total DNA was extracted from in vitro banana leaves using a CTAB-based method (Stewart and Via 1993). PCR was used to confirm the presence of the *gfp* transgene (primers *gfp*-F/*gfp*-R), absence of the pMF1 vector backbone (*trf*A-F/*trf*A-R), recombination positive (*FW1*-F/*gfp*-REC-R and *FW2*-F/*gfp*-REC-R), recombination negative (*FW3*-F/*gfp*-REC-R), excision of selection genes (*cod*A/*npt*II-F/*cod*A/*npt*II-R), absence of residual *Agrobacterium* (*virG*-F/*virG*-R) and excision of recombinase gene (*REC:LBD*-F/*REC:LBD*-R). Approximately 100 ng of DNA was used as the template in a PCR containing GoTaq^®^ Green PCR Master Mix system (Promega) and 10 ρmol of each primer pair (Table 1). PCRs were cycled using the following conditions: 94 °C for 2 min, followed by 25 cycles of 94 °C for 30 s/55–60 °C for 30 s/72 °C for 1 min and a final extension of 72 °C for 2 min. PCR amplicons were electrophoresed through a 1% agarose gel.

**Table 1:** List of primer pairs used for genotyping and the anticipated amplicon size

Primer name	Primer sequence (5′ → 3′)	Amplicon size (bp)
*gfp*-F	ATGGTGAGCAAGGGCGAGGAGCTGTT	719
*gfp*-R	TTTACTTGTACAGCTCGTCCATGCC	
*trf*A-F	GCGAGGAACTATGACGACCA	325
*trf*A-R	CCACACCAGTTCGTCATCGT	
*FW1*-F	ACGGATCTACGATTTGATGA	976 (excised)
*gfp*-REC-R	GGGTCTTGTAGTTGCCGTCGTCCTT	
*FW2*-F	GTGGTGTAAACGGATCTACGATTTGA	985 (excised)
*gfp*-REC-R	GGGTCTTGTAGTTGCCGTCGTCCTT	
*FW3*-F	AACCTGCTCTGCTTTGCTCCTGATC	1736 (unexcised)
*gfp*-REC-R	GGGTCTTGTAGTTGCCGTCGTCCTT	
*cod*A/*npt*II -F	AAGGTGATTGCCAGCACACA	511
*cod*A/*npt*II -R	TACGTGCTCGCTCGATGCGA	
*virG*-F	GCCGGGGCGAGACCATAGG	606
*virG*-R	CGCACGCGCAAGGCAACC	
*REC:LBD*-F	ACCTCATCGAAGCTAGAACCACTC	510
*REC:LBD*-R	CCTTCCTCACGAGCGGCAGACC	

### Cloning and sequencing of the recombination footprint

PCR amplicons were excised from agarose and purified using a High Pure PCR Product Purification kit (Promega) according to manufacturer’s guidelines. Products were ligated into pGEM^®^-T Easy vector and cloned into *E. coli* by the heat shock method. Plasmid DNA was isolated by alkaline lysis and Sanger sequenced with M13 forward and reverse primers by Macrogen Inc. (South Korea).

### Digital droplet (dd)PCR for copy number estimation

Total DNA was extracted from in vitro banana leaves using a GenElute Plant Genomic DNA miniprep Kit (Sigma). Reaction mixes were prepared using the ddPCR™ Supermix for Probes (no dUTP) cocktail (BioRad) and PCR cycling parameters carried out according to the manufacturer’s instructions. Primers and probes used for amplification and detection are listed in Table 2. Total DNA (1 µg) was digested with 10 U of HindIII by incubation at 37 °C for 1 h then heat deactivated at 80 °C for 20 min. The reaction mix was loaded into the sample wells of a DG8™ Cartridge for QX200 Droplet Generator followed by 70 μL of Droplet Generation Oil, according to the QX200 Droplet Generator Instruction Manual (BioRad). The banana *mcm*5 gene was used as a single copy reference gene (GenBank Accession: XM_009383287.2).

**Table 2:** List of primers and probes used for ddPCR copy number estimation

Primer name	Primer sequence (5′ → 3′)	Amplicon size (bp)	Probe sequence
dd*gfp*-F	GCACAAGCTGGAGTACAACTA	98	5′/HEX/AGCAGAAAGA/ZEN/ACGGCATCAAGGTGA/3IABkFQ3′
dd*gfp*-R	TGTTGTGGCGGATCTTGAA		
*mcm*-F	AGATGCTATAGATGCCCATAAC	100	5′/56-FAM/CCAGGCACA/ZEN/ATAGTCTGAACCAGGT/3IABkFQ3′
*mcm*-R	GGAGAGCTGCCAAGAAATA		

### Confirmation of plant sensitivity to kanamycin and 5-FC

Plants representing wild-type, unexcised (pMF1-GFP without DEX/5-FC treatment) and excised (pMF1-GFP with DEX/5-FC treatment) were sub-cultured onto (i) MS media only, (ii) MS media containing kanamycin (100 mg/L) and (iii) MS media containing 5-FC (150 mg/L). Plants were maintained in tissue culture for 2 weeks under standard growth conditions.

### Visualization of GFP expression

GFP expression in whole plants and tissues was visualized under blue light and photographed using a blue light box and a Canon d-SLR camera with an orange filter.

## Results

### Banana transformation and DEX/5-FC chemical treatments

Banana transformation and regeneration is a lengthy process that occurs through five sequential phases (i) *Agrobacterium*-mediated transformation, (ii) cell division and antibiotic selection, (iii) somatic embryo formation, (iv) plant regeneration and (v) plant rooting. Two *Agrobacterium* strains (LBA4404 and AGL1) were used to transform two cultivars of Cavendish banana (Williams and Grand Naine) with the pMF1-GFP vector in order to assess whether virulence of the bacteria influences the number of T-DNA copies integrated into the banana genome, the prevalence of unwanted vector backbone and ultimately the effectiveness of DEX-mediated recombination and 5-FC-based negative selection.

Each banana cultivar was transformed using either *Agrobacterium* strain i.e. LBA4404:Williams, LBA4404:Grand Naine, AGL1:Williams, and AGL1:Grand Naine. Half of the transformed cells from each combination were processed through the standard transgenic banana regeneration protocol whereby plants were regenerated under kanamycin selection but without DEX/5-FC treatment. These plants represented the “unexcised” transgenic events and served as control lines for both regeneration efficiency and copy number analysis. The other half of the transformed cells were processed through the DEX/5-FC treatment in order to remove the selectable marker gene. In this case, DEX/5-FC treatments were targeted to the pre-embryo stage (3 months post-transformation) which provided ample time for kanamycin antibiotic selection of the transformed cells but was early enough in the regeneration process to minimize the chance of producing chimeric plant tissues. For DEX treatment, cells were transferred to liquid BL media supplemented with 100 µM DEX and the cell suspensions were maintained for a period of either 5 or 10 days depending on the *Agrobacterium* strain used for transformation. Cells were transferred to liquid for this period in order to maximize exposure to DEX and to simulate the normal subculturing cycle of embryogenic cell suspensions. Importantly at this point, transformed cells from a single plate were pooled and, despite the fact that up to 4 individual plants were regenerated from each cell pool, only one plant was ultimately selected as a representative transgenic event. Cells still containing the *cod*A gene fusion were negatively selected immediately after DEX-mediated recombination, by supplementing M3 media with 5-FC at a concentration of 150 mg/L and maintaining low levels of DEX (1 µM) in the media. 5-FC and DEX were retained in the media (at the same levels) throughout all subsequent subculturing to the point of plant regeneration and rooting. From the total cell pool, between 16–20 plants were regenerated using this strategy for each *Agrobacterium* strain:banana cultivar. These plants represented the “excised” transgenic events in which the selectable marker gene had potentially been removed. Based on superior growth characteristics, 26 to 30 independent “unexcised” and 5 “excised” events representing each *Agrobacterium* strain:banana cultivar transformation were selected for further analysis,

### PCR characterization of transgenic events

**Fig. 2:**
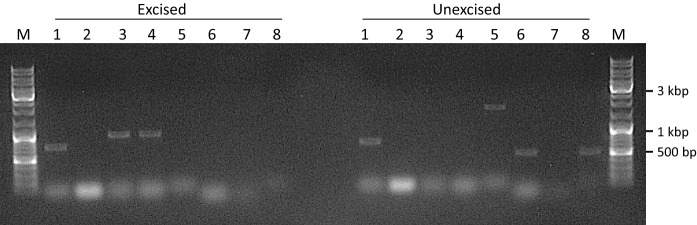
PCR-based genotyping of representative excised and unexcised events. Assorted primer sets were used in a PCR to amplify target sequences from a plant representing an excised event and an unexcised event. Amplicons were electrophoresed through a 1.0% agarose gel. Primer sets include (1) *gfp* transgene: *gfp*-F/*gfp*-R, (2) *trf*A vector backbone: *trf*A-F/*trf*A-R, (3) FW1 to prove recombination: *FW1*-F/*gfp*-R, (4) *FW2* to prove recombination: *FW2*-F/*gfp*-R, (5) FW3 to prove non-recombination: *FW3*-F/*gfp*-R, (6) *cod*A*:npt*II gene fusion: *cod*A*:npt*II-F/*cod*A*:npt*II-R, (7) *vir*G from *Agrobacterium*: *vir*G-F/*vir*G-R, and (8) *REC:LBD* gene fusion: *REC:LBD*-F/*REC:LBD*-R. M = GeneRuler DNA Ladder (Thermo Scientific). All primer sequences and expected amplicon sizes are described in Table 1

Plants were genotyped by PCR in order to confirm (i) presence of the *gfp* transgene, (ii) absence of pMF1 vector backbone sequence (iii) excision at the recombination site, (iv) excision of selection genes (absence of the *cod*A:*npt*II gene fusion), (v) absence of residual *Agrobacterium*, and (vi) excision of the recombinase gene (absence of the *REC:LBD* gene fusion). To do this, primer sets were strategically designed spanning these genetic features to differentiate between successful/unsuccessful excision and recombination events. All twenty excised events (5 plants representing each *Agrobacterium* strain:banana cultivar transformation) were PCR screened using these primer sets. An unexcised plant, wild-type plant, and positive and negative template controls were also screened with these primer sets (“Appendix 1”). Figure 2 is a representative agarose gel of PCR amplicons obtained from both excised and unexcised events. For excised and unexcised events, PCR amplicons were obtained for the *gfp* reporter confirming the presence of the transgene. No PCR amplicons were obtained using the *vir*G primer set, indicating these plants were free from residual contaminating *Agrobacterium*. For the unexcised event, PCR amplicons of the anticipated size were evident for the *cod*A:*npt*II gene fusion, the *REC:LBD* gene fusion, and the *FW3*/*gfp*-REC-R primer sets, but not the *trf*A vector backbone or *FW1*/*gfp*-REC-R and *FW2*/*gfp*-REC-R primer sets. In contrast, for the excised plant, no PCR products were obtained for the *cod*A:*npt*II gene fusion, the *REC:LBD* gene fusion, the *trf*A vector backbone and the *FW3*/*gfp*-REC-R primer set, but a product was amplified with the *FW1*/*gfp*-REC-R and *FW2*/*gfp*-REC-R primer sets. Importantly, the *FW1*/*gfp*-REC-R and *FW2*/*gfp*-REC-R primer sets only amplify a product when excision and recombination have occurred whereas the *FW3*/*gfp*-REC-R primer set only amplify a product when recombination and excision have not occurred. This would suggest that the excised line is free of the *cod*A:*npt*II and *REC*-*LBD* gene sequences and that recombination at the 82 bp footprint had indeed occurred. Using this strategy, all twenty excised events were proven to be free of the selectable marker gene (results not shown). However, when using primers designed to amplify the *trf*A gene in the vector backbone, PCR products were amplified in 10 out of 20 excised plants. Of the *Agrobacterium* strain:banana cultivars tested, only plants representing AGL1:Williams were 100% free of backbone with non T-DNA detectable in 40–80% of plants representing other combinations. This finding suggests the prevalence of integrated vector backbone is relatively high despite successful excision.

### Verification of the recombination site in excised lines

In order to verify the 82 bp recombinase footprint had been reconstituted following DEX-mediated excision, the PCR product obtained using the *FW1*/*gfp*-REC-R primer set was ligated into pGEM^®^-T Easy vector and transformed into *E. coli*. Clones were obtained for a single copy excised plant representing each *Agrobacterium* strain:banana cultivar. Three clones representing each plant were Sanger sequenced and the reads aligned with the anticipated recombination sequence. All traces matched the exact sequence of the footprint and adjacent sequences verifying precise recombination and excision in these plants (“Appendix 2”).

### Copy number estimation

To determine whether the choice of *Agrobacterium* strain influences transgene copy number and whether the success of marker gene removal is biased towards those cells that contain fewer T-DNA insertions, we used ddPCR to estimate the number of transgene integrations in a large number of regenerated plants (Table 3). Between 26 and 30 unexcised plants representing each *Agrobacterium* strain:banana cultivar transformation were tested for copy number. For all combinations, except AGL1:Grand Naine, copy number was low, averaging between 2 to 5 *gfp* transgene integrations. In contrast, Grand Naine plants obtained by AGL1 transformation were particularly high averaging 13 copies. All 20 excised events were also assessed for the number of *gfp* transgene integrations. In these cases, all plants showed very low copy number averaging 1–2 copies, except the AGL1:Grand Naine combination that averaged 3 *gfp* transgene copies per plant.

**Table 3:** Transgene copy number estimation in excised and unexcised events using ddPCR

*Agrobacterium* strain/banana cultivar	Number of copies	Number of lines
LBA4404:Grand Naine unexcised	1	6
	2	23
	3	0
	More than 3	0
	Average = 2	Total = 29
AGL1:Grand Naine unexcised	1	4
	2	2
	3	0
	More than 3	20
	Average = 13	Total = 26
LBA4404:Williams unexcised	1	21
	2	0
	3	7
	More than 3	2
	Average = 2	Total = 30
AGL1:Williams unexcised	1	8
	2	6
	3	4
	More than 3	11
	Average = 5	Total = 29
LBA4404:Grande Naine excised	1	5
	2	0
	3	0
	More than 3	0
	Average = 1	Total = 5
AGL1:Grand Naine excised	1	1
	2	2
	3	1
	More than 3	1
	Average = 3	Total = 5
LBA4404:Williams excised	1	2
	2	3
	3	0
	More than 3	0
	Average = 2	Total = 5
AGL1:Williams excised	1	4
	2	1
	3	0
	More than 3	0
	Average = 1	Total = 5

### GFP expression and exposure to kanamycin and 5-FC

**Fig. 3:**
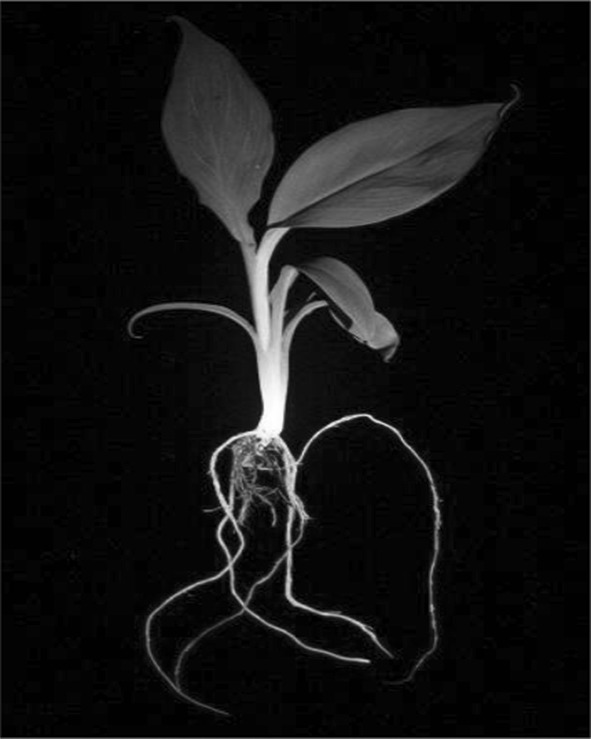
GFP expression in a marker-free banana plant. GFP expression in tissues of a representative selectable marker gene-free whole banana plant (LBA4404:Williams). The plant was visualized under blue light and photographed using a blue light box and a Canon d-SLR camera with an orange filter

**Fig. 4:**
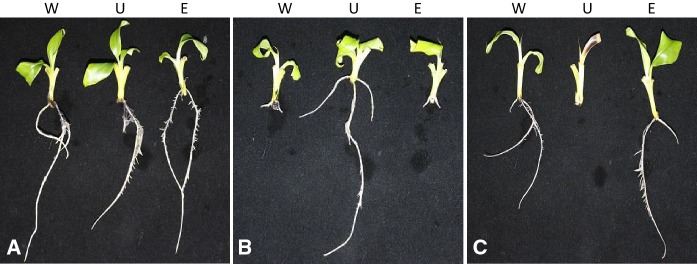
Confirmation of plant sensitivity to kanamycin and 5-FC. Wild-type (W), unexcised (U) and excised (E) banana plants were subcultured onto (A) MS media only, (B) MS media containing kanamycin (100 mg/L) and (C) MS media containing 5-FC (150 mg/L). Plants were grown for 2 weeks and photographed

GFP expression in the excised events was visualized under blue light and all 20 plants displayed strong green fluorescence in the leaves, pseudostem and roots (Fig. 3). To demonstrate the effective elimination of the *npt*II and *cod*A selection genes and, therefore, their sensitivity to kanamycin and tolerance to 5-FC, respectively, an excised plant was grown in MS media alone, MS media containing kanamycin (100 mg/L) and MS media containing 5-FC (150 mg/L). As controls, a wild-type and an unexcised plant were included in the same treatments. After 2 weeks in tissue culture, all plants grown on MS media alone appeared healthy and produced an abundant root system (Fig. 4A). When grown on MS media containing kanamycin, only the unexcised plant proliferated whereas the wild-type and excised event appeared stunted and did not produce roots (Fig. 4B). In contrast, when grown on MS media containing 5-FC, only the wild-type and excised event thrived whereas the unexcised event was significantly stunted, produced no roots (Fig. 4C) and later died. These findings suggested that only the unexcised plant contained the *npt*II and *cod*A genes, that confer resistance to kanamycin and susceptibility to 5-FC, respectively.

## Discussion

Future proofing the Cavendish banana against current and emerging disease threats will be reliant on transgenic technologies. For instance, the apparent absence of a BBTV resistance (R) gene in the banana gene pool coupled with the high level of sterility in almost all domesticated female banana parents essentially precludes conventional breeding as an option for generating BBTV resistance. However, using a transgenic RNAi approach, whereby hairpin RNA targeting the BBTV Master replication initiation gene was over-expressed in transgenic plants (Shekhawat et al. 2012; Elayabalan et al. 2013), virus resistance was achieved.

In the case of banana transformation, the use of a selectable marker gene to assist in the proliferation and regeneration of cells containing the gene of interest is paramount. Two antibiotic resistance genes have been used primarily for this purpose, the *npt*II and *hph* genes, conferring resistance to certain aminoglycosides (including kanamycin) and the aminocyclitol inhibitor hygromycin B, respectively (Nap et al. 1992; Waldron et al. 1985; van den Elzen et al. 1985). These genes represent the two most commonly used selectable markers in transgenic crops and there is an extensive body of evidence that these products pose little to no risk to the environment or human health (Miki and McHugh 2004). In fact, NPTII has been approved by the US Food and Drug Administration (FDA) as a food additive and use of the *npt*II selectable marker gene in genetically modified plants was deemed to pose no risk to human health (WHO 1993). Despite the scientific evidence, public concerns over gene flow, horizontal transfer and toxicity persist. Accordingly, the production of transgenic plants free of the selectable marker gene would greatly improve public opinion and simplify the regulatory process towards the deregulation of genetically modified crops. From a scientific perspective, it would also allow for the serial improvement of elite GM banana with superior agro-traits. Being a vegetatively propagated crop, the option to breed away from the selection gene is not applicable for GM banana and, therefore, excision of the selectable marker gene is a convenient alternative.

To date, only one recombination/excision system has been successfully adapted to banana, the Cre-lox platform derived from the *E. coli* bacteriophage P1. Chong-Pérez et al. (2012, 2013) used two approaches to control the expression of the *cre* gene and therefore recombination of the *lox* sites. In the first study, the *cre* recombinase gene was placed under the control of a heat shock-inducible promoter and the *cre* and *hph* genes were placed between two *lox* sites. The transgene of interest was inserted outside of the *lox* recombination sites. Transient heat shock treatment of primary transgenic embryos was sufficient to induce *cre* expression and excise both the *cre* and *hph* genes, with excision efficiency reaching between 40 and 60%. In the second approach, an embryo-specific promoter was used to direct *cre* expression. This strategy resulted in excision of the *cre* and *hph* genes in mature somatic embryos with 34% of regenerated plants devoid of the *hph* gene. While these studies demonstrated the feasibility of using inducible or developmentally controlled promoters to mediate DNA excision in banana, in both cases, the gene of interest remaining after excision was in fact the *npt*II gene, thus, these plants were not free of the selectable marker gene.

In the present study, we adopted the R-Rs recombinase platform developed originally for plant systems by Onouchi et al. (1995) and Sugita et al. (2000), and later adapted into a sequential double selection vector, pMF1, by Schaart et al. (2004). Unlike the Cre-lox system described above, the Recombinase:LBD fusion is constitutively expressed and its activity controlled by steroid-induced translocation into the nucleus, thereby obviating the need for highly regulated plant promoters. This system has been successfully developed for the production of marker-free plants including strawberry, apple and pear (Schaart et al. 2004; Vanblaere et al. 2011; Righetti et al. 2014; Krens et al. 2015; Timerbaev et al. 2019). Using two different strains of *Agrobacterium* for gene transfer we established a chemical treatment and regeneration protocol for the elimination of the selectable marker gene in two Cavendish banana cultivars. The number of excised events obtained using this regime was lower than that obtained using standard transformation, however, this likely reflects the fact that a large number of transformed cells were pooled for liquid DEX treatment and this cell pool was subsequently considered as a single transformation event to exclude the possibility of regenerating multiple biological replicates of the same transgenic event. If cells were treated with DEX on solid media and individual transformants separated upon plantlet formation it would greatly increase the number of independent events obtained. Alternatively, all plants regenerated using the current protocol could be fully genotyped to identify independent events.

From a regulatory perspective, it is desirable for a genetically modified plant to contain a single copy of the transgene and be free of extraneous DNA, particularly vector backbone. In this study, we showed that *Agrobacterium* strains LBA4404 and AGL1 differ in their capacity to deliver T-DNA into two Cavendish cultivars, Williams and Grand Naine. In general, LBA4404 appeared to be the less virulent of the two strains averaging 2 transgene copies in unexcised events from both cultivars. Contrastingly, AGL1 generated very high average copy numbers of 5 and 13 in Williams and Grand Naine, respectively. In the case of those events that had undergone DEX/5-FC treatments, plants generally contained 1–2 transgene copies, except in the case of AGL1:Grand Naine which averaged 3 copies. This would suggest the intrinsic properties of DEX-mediated recombination and 5-FC negative selection may favour those events with low copy number over those containing multiple T-DNA integrations or perhaps with complex multimeric arrangements. PCR screening of the excised events demonstrated the absence of the *cod*A:*npt*II and *REC:LBD* gene fusions and sequencing across the recombination site verified the integrity of 82 bp recombinase footprint. Further, these excised lines were susceptible to the effects of kanamycin but unaffected by exposure to 5-FC. Together, this is strong evidence that these *gfp*-expressing transgenic events represent the first true selectable marker gene-free Cavendish banana plants. However, 50% of excised events were proven to contain vector backbone inserted in their genome which is approximately two-fold higher than that previously reported for non T-DNA integration following *Agrobacterium*-mediated transformation of banana (Pérez-Hernández et al. 2006). This likely represents border skipping by the VirD2 endonuclease during T-DNA transfer (Gelvin 2017) which could perhaps be minimized by duplicating the left T-DNA border (Kuraya et al. 2004) and/or including a lethal gene in the vector backbone (Hanson et al. 1999); two strategies that have proven to significantly reduce the integration frequency of extraneous plasmid sequence into plant genomes during *Agrobacterium*-mediated transformation.

The strategy described in this paper will be particularly useful for the development of cisgenic bananas and provides the opportunity for serial transformation of elite transgenic events into the future. The pMF1 recombination system also ensures a very high proportion of regenerated banana plants are single copy events, an aspect particularly useful from a scientific and regulatory perspective. With additional refinements to the platform we envision that the system can be further improved. To this end, we are currently assessing a rapid antibiotic selection approach that significantly reduces the time to regenerate an excised banana plant and evaluating an alternative means of DEX treatment to greatly increase the number of excised events generated per transformation. Further, we anticipate subtle modifications to the pMF1 vector will improve the likelihood of regenerating excised events free of extraneous vector sequence.

## Figures and Tables

**Fig. 5:**
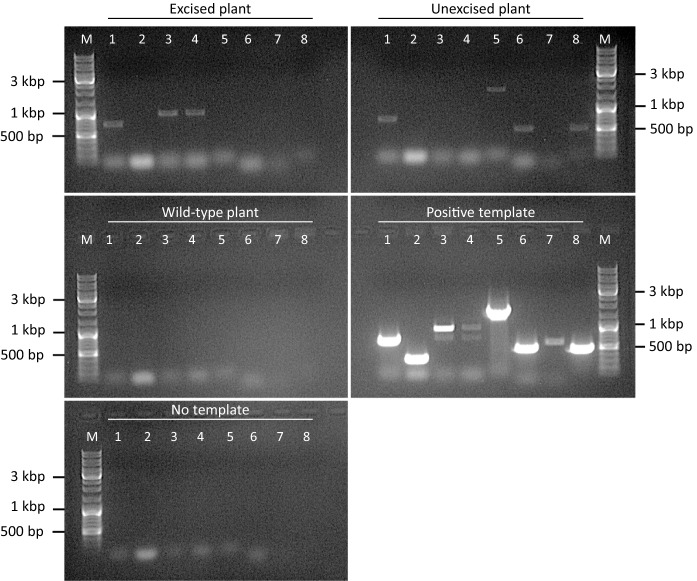
PCR-based genotyping of an excised and unexcised event including controls. Assorted primer sets were used in a PCR to amplify target sequences from a plant representing an excised event and an unexcised event. PCRs on a wild-type plant, positive template and no template were included as controls. Amplicons were electrophoresed through a 1.0% agarose gel. Primer sets include (1) *gfp* transgene: *gfp*-F/*gfp*-R, (2) *trf*A vector backbone: *trf*A-F/*trf*A-R, (3) FW1 to prove recombination: *FW1*-F/*gfp*-R, (4) *FW2* to prove recombination: *FW2*-F/*gfp*-R, (5) FW3 to prove non-recombination: *FW3*-F/*gfp*-R, (6) *cod*A*:npt*II gene fusion: *cod*A*:npt*II-F/*cod*A*:npt*II-R, (7) *vir*G from *Agrobacterium*: *vir*G-F/*vir*G-R, and (8) *REC:LBD* gene fusion: *REC:LBD*-F/*REC:LBD*-R. M = GeneRuler DNA Ladder (Thermo Scientific). All primer sequences and expected amplicon sizes are described in Table 1

**Fig. 6:**
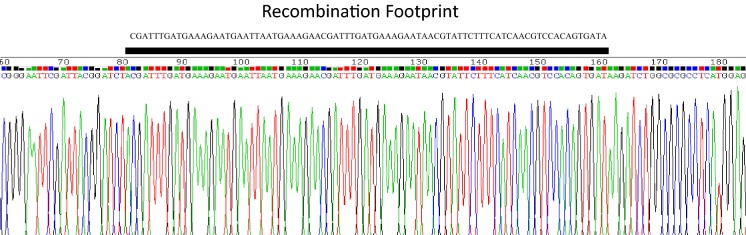
Verification of precise recombination following DEX treatment. The recombination footprint in an excised, single transgene copy banana plant representing a LBA4404:Williams transformation was PCR amplified using the *FW1*/*gfp*-REC-R primer set. The amplicon was cloned and Sanger sequenced. Sequence reads were aligned to the anticipated footprint following DEX-mediated recombination

## Appendix 1

See Fig. 5.

## Appendix 2

See Fig. 6.
